# One-carbon metabolism, cognitive impairment and CSF measures of Alzheimer pathology: homocysteine and beyond

**DOI:** 10.1186/s13195-017-0270-x

**Published:** 2017-06-17

**Authors:** Loïc Dayon, Seu Ping Guiraud, John Corthésy, Laeticia Da Silva, Eugenia Migliavacca, Domilė Tautvydaitė, Aikaterini Oikonomidi, Barbara Moullet, Hugues Henry, Sylviane Métairon, Julien Marquis, Patrick Descombes, Sebastiano Collino, François-Pierre J. Martin, Ivan Montoliu, Martin Kussmann, Jérôme Wojcik, Gene L. Bowman, Julius Popp

**Affiliations:** 1 Nestlé Institute of Health Sciences, École Polytechnique Fédérale de Lausanne (EPFL) Innovation Park, Bâtiment H, 1015 Lausanne, Switzerland; 2 Old Age Psychiatry, Department of Psychiatry, Centre Hospitalier Universitaire Vaudois (CHUV), Lausanne, Switzerland; 3 Department of Laboratories, Centre Hospitalier Universitaire Vaudois (CHUV), Lausanne, Switzerland; 4 Quartz Bio, Geneva, Switzerland; 5 Present address: Liggins Institute, University of Auckland, Auckland, New Zealand

**Keywords:** Alzheimer’s disease, Cognition, CSF, Homocysteine, Metabolomics, One-carbon metabolism, *S*-adenosyl-L-homocysteine, Tau

## Abstract

**Background:**

Hyperhomocysteinemia is a risk factor for cognitive decline and dementia, including Alzheimer disease (AD). Homocysteine (Hcy) is a sulfur-containing amino acid and metabolite of the methionine pathway. The interrelated methionine, purine, and thymidylate cycles constitute the one-carbon metabolism that plays a critical role in the synthesis of DNA, neurotransmitters, phospholipids, and myelin. In this study, we tested the hypothesis that one-carbon metabolites beyond Hcy are relevant to cognitive function and cerebrospinal fluid (CSF) measures of AD pathology in older adults.

**Methods:**

Cross-sectional analysis was performed on matched CSF and plasma collected from 120 older community-dwelling adults with (*n* = 72) or without (*n* = 48) cognitive impairment. Liquid chromatography-mass spectrometry was performed to quantify one-carbon metabolites and their cofactors. Least absolute shrinkage and selection operator (LASSO) regression was initially applied to clinical and biomarker measures that generate the highest diagnostic accuracy of a priori-defined cognitive impairment (Clinical Dementia Rating-based) and AD pathology (i.e., CSF tau phosphorylated at threonine 181 [p-tau181]/β-Amyloid 1–42 peptide chain [Aβ_1–42_] >0.0779) to establish a reference benchmark. Two other LASSO-determined models were generated that included the one-carbon metabolites in CSF and then plasma. Correlations of CSF and plasma one-carbon metabolites with CSF amyloid and tau were explored. LASSO-determined models were stratified by apolipoprotein E (*APOE*) ε4 carrier status.

**Results:**

The diagnostic accuracy of cognitive impairment for the reference model was 80.8% and included age, years of education, Aβ_1–42_, tau, and p-tau181. A model including CSF cystathionine, methionine, *S*-adenosyl-L-homocysteine (SAH), S-adenosylmethionine (SAM), serine, cysteine, and 5-methyltetrahydrofolate (5-MTHF) improved the diagnostic accuracy to 87.4%. A second model derived from plasma included cystathionine, glycine, methionine, SAH, SAM, serine, cysteine, and Hcy and reached a diagnostic accuracy of 87.5%. CSF SAH and 5-MTHF were associated with CSF tau and p-tau181. Plasma one-carbon metabolites were able to diagnose subjects with a positive CSF profile of AD pathology in *APOE* ε4 carriers.

**Conclusions:**

We observed significant improvements in the prediction of cognitive impairment by adding one-carbon metabolites. This is partially explained by associations with CSF tau and p-tau181, suggesting a role for one-carbon metabolism in the aggregation of tau and neuronal injury. These metabolites may be particularly critical in *APOE* ε4 carriers.

**Electronic supplementary material:**

The online version of this article (doi:10.1186/s13195-017-0270-x) contains supplementary material, which is available to authorized users.

## Background

The methionine, purine, and thymidylate cycles together encompass one-carbon metabolism in the cytosol [1]. Both genetic (i.e., polymorphisms in *MTHFR*) and environmental factors influence one-carbon metabolism; however, genetic factors are thought to play a minor role, whereas dietary intake of folate, vitamin B_6_, and vitamin B_12_ explains 35% of the variation [2].

The most-studied biomarker of one-carbon metabolism is homocysteine (Hcy), a sulfur-containing amino acid generated by the metabolism of methionine [3]. Inherited deficiencies in cystathionine synthase, methionine synthase, or methylene-tetrahydrofolate reductase enzymes causing homocystinuria and severe atherosclerotic plaques were discovered in children in the mid-20th century [4]. More recently, hyperhomocysteinemia has been identified as a risk factor for a multitude of conditions, including cognitive decline and Alzheimer dementia [5–10]. Elevated plasma Hcy is linked with deficiency in vitamins B_6_, B_12_, and folate, as well as oxidative damage [11, 12]. However, Hcy-lowering trials with B vitamins have met with mixed results that have dampened enthusiasm for targeting Hcy in the prevention of cognitive decline or Alzheimer disease (AD) progression [13]. There may be several explanations for this inconsistency, but one often overlooked is that emphasis on Hcy specifically may be insufficient in magnitude of effect under the constraints of current clinical trial designs. A role for other participants in the one-carbon cycle that are highly interactive with Hcy may have been underappreciated. For example, *S*-adenosylmethionine (SAM), *S*-adenosyl-L-homocysteine (SAH), and 5-methyltetrahydrofolate (5-MTHF) balance may operate on several mechanisms that support cognitive function, ranging from epigenetic modulation of synaptic function in the hippocampus to methylation reactions in the liver that convert phosphatidylethanolamine to phosphatidylcholine and facilitate the delivery of essential fatty acids (i.e., docosahexaenoic acid) to the plasma and the brain [14–18].

The ε4 allele of apolipoprotein E (*APOE*) gene is a well-known and major genetic risk factor for AD [19]. Several recent studies have shown the interaction of *APOE* genotype and environmental factors, such as diet, on the risk of dementia and AD [20]. Nutrient intake effect on cognitive function might be influenced by *APOE* status, and the association between B_12_ and cognitive function was indeed shown to be stronger in *APOE* ε4 carriers [21]. The effects of vitamin B_12_ and Hcy on gray matter volume may also be influenced by *APOE* genotype in AD [22]. The clear interaction between Hcy, its cofactors, and *APOE* remains to be more broadly investigated.

Metabolomics is an approach with the potential to target and identify perturbations in specific pathways of interest by quantifying a wide range of biochemical compounds in tissue [23]. We leveraged this approach to test the hypothesis that comprehensive assessment of one-carbon metabolism in cerebrospinal fluid (CSF) and plasma would better explain cognitive impairment and CSF measures of AD pathology in older adults.

## Methods

### Study population

The participants with cognitive impairment were recruited among outpatients who were referred to the memory clinics, departments of psychiatry, and the Leenaards Memory Center, Department of Clinical Neurosciences, University Hospitals of Lausanne (Switzerland). Cognitively intact participants were recruited from the community through advertisement or among the spouses of memory clinic patients*.* In total, 120 subjects were enrolled in the biomarker study.

### CSF and plasma sample collection

Venous and lumbar punctures were performed between 8:30 and 9:30 a.m. after overnight fasting. For lumbar puncture, a standardized technique with a 22-gauge “atraumatic” spinal needle and the subject in a sitting or lying position was applied [24]. A volume of 10–12 ml of CSF was collected in polypropylene tubes. CSF samples were centrifuged, frozen in aliquots, and stored at −80 °C before further use. Blood was drawn into ethylenediaminetetraacetic acid-containing vacutainers. After 20–30 minutes on ice, the tubes were centrifuged. Plasma samples were aliquoted in polypropylene tubes and stored at −80 °C.

### CSF β-amyloid 1–42 peptide chain, tau, tau phosphorylated at threonine 181, and *APOE* ε4 genotyping

The measurements were performed using commercially available enzyme-linked immunosorbent assay kits and TaqMan assays (Applied Biosystems, Foster City, CA, USA) as described in Additional file 1: Supplementary Methods.

### Targeted metabolomics of one-carbon metabolism

Liquid chromatography-tandem mass spectrometry (LC-MS/MS) was performed to measure absolute concentrations of metabolites and cofactors of one-carbon metabolism in CSF and blood plasma as described previously [23] (*see* Additional file 1: Supplementary Methods).

### Primary outcome measures

#### Cognitive impairment

Diagnosis of mild cognitive impairment (MCI) or dementia was based on neuropsychological and clinical evaluation and made by a consensus conference of psychiatrists and/or neurologists and neuropsychologists prior to subject inclusion. The participants had no major psychiatric or neurological disorders, nor did they have substance abuse or severe or unstable physical illness that might contribute to cognitive impairment. Magnetic resonance imaging and computed tomographic scans were used to exclude cerebral pathologies possibly interfering with the cognitive performance. Subjects known to take folate and B-vitamin supplementation were excluded from the study. MCI was diagnosed according to widely used consensus recommendations [25]. Subjects with MCI had memory impairment [26] and/or impairment in another cognitive domain such as executive tasks, as well as a Clinical Dementia Rating (CDR) [27] score of 0.5. The diagnosis of probable AD dementia was defined according to the clinical diagnostic criteria for probable dementia due to AD according to the recommendations of the National Institute on Aging and the Alzheimer’s Association [28] as well as the criteria of the *Diagnostic and Statistical Manual of Mental Disorders, Fourth Edition*, for dementia of the Alzheimer type [29] with a CDR of 1. In the present study, we had only nine subjects with AD. Because there is a clinical continuum between MCI and mild dementia, and because the participants with cognitive impairment were patients from memory clinics recruited in the same way regardless of MCI or mild dementia classification, these subjects were collapsed with the MCI group and labeled as cognitively impaired. The participants without cognitive impairment had no history or evidence of cognitive deficits and required a CDR of 0. We therefore defined two categories of subjects on the basis of their CDR (i.e., CDR 0 or >0 [i.e., 0.5 or 1]) (Table 1).

**Table 1:** Demographics and clinical characteristics

	All (*n* = 120)	Cognitively normal (CDR 0) (*n* = 48)	Cognitively impaired (CDR 0.5 or 1) (*n* = 72)
Age, years	70.4 (7.9)	66.0 (7.4)	73.3 (6.9)^a^
Male sex, *n* (%)	43 (35.83%)	17 (35.42%)	26 (36.11%)
Education, years	12.4 (2.6)	13.2 (2.3)	11.8 (2.7)^a^
MMSE score, mean	26.9 (3.1)	28.5 (1.4)	25.9 (3.5)^a^
*APOE* ε4 carrier, *n* (%)	37 (30.83%)	11 (22.92%)	26 (36.11%)^a^
CSF parameters			
Aβ_1–42_, pg/ml	847.4 (265.1)	957.4 (194.0)	774.0 (281.5)^a^
tau, pg/ml	371.3 (278.6)	221.5 (82.9)	471.1 (316.6)^a^
p-tau181, pg/ml	62.0 (35.2)	45.9 (13.3)	72.7 (40.9)^a^
p-tau181/Aβ_1–42_	0.088 (0.082)	0.049 (0.015)	0.114 (0.097)^a^
Albumin index^b^	6.1 (2.4)	5.3 (1.9)	6.6 (2.5)^a^
Choline, μM	2.7 (0.5)	2.6 (0.4)	2.8 (0.6)^a^
Cystathionine, nM	35.8 (17.0)	34.4 (13.8)	36.7 (18.9)
Methionine, μM	3.6 (0.8)	3.3 (0.5)	3.9 (0.9)^a^
*S*-adenosylhomocysteine, nM	15.1 (5.8)	13.5 (4.3)	16.1 (6.3)^a^
*S*-adenosylmethionine, nM	183.0 (42.0)	188.2 (41.1)	179.4 (42.5)
Serine, μM	27.1 (4.7)	25.7 (4.1)	28.0 (4.8)^a^
Cysteine, μM	1.2 (0.4)	1.2 (0.4)	1.3 (0.4)
5-Methyltetrahydrofolate, nM	45.1 (12.5)	47.2 (10.9)	43.8 (13.4)
Plasma parameters			
Cystathionine, nM	252.3 (232.4)	267.5 (290.0)	242.1 (185.9)
Glycine, μM	234.5 (60.6)	235.9 (59.7)	233.6 (61.5)
Methionine, μM	19.7 (3.6)	19.4 (2.9)	19.9 (4.1)
*S*-adenosylhomocysteine, nM	20.9 (9.1)	18.3 (6.3)	22.7 (10.2)^a^
*S*-adenosylmethionine, nM	71.9 (20.7)	67.6 (17.7)	74.7 (22.2)
Serine, μM	115.3 (19.9)	111.5 (16.9)	117.9 (21.3)
Cysteine, μM	158.3 (20.3)	150.7 (18.4)	163.3 (20.1)^a^
Homocysteine, μM	5.6 (1.6)	5.3 (1.5)	5.8 (1.7)

*Abbreviations: Aβ* β-Amyloid, *APOE* Apolipoprotein E, *CDR* Clinical Dementia Rating, *CSF* Cerebrospinal fluid, *MMSE* Mini Mental State Examination, *p-tau181* Tau phosphorylated at threonine 181

Data are given as means with SD within parentheses, unless indicated otherwise

^a^Statistically different (*p* ≤ 0.05) from CDR 0 using *t* tests for continuous variables and binomial proportion tests for categorical variables

^b^CSF albumin index = [CSF albumin]/[serum albumin] × 100

#### CSF profiles of AD pathology

Subjects were also classified into two groups on the basis of their CSF tau phosphorylated at threonine 181 (p-tau181)/β-amyloid 1–42 peptide chain (Aβ_1–42_) ratio: “low” when p-tau181/Aβ_1–42_ was ≤0.0779 or “high” when p-tau181/Aβ_1–42_ was >0.0779, considered as negative and positive CSF profiles of AD pathology, respectively. This threshold optimized the Youden index [30] of the receiver operating characteristic (ROC) curve for the prediction of CDR categories (CDR 0 versus CDR >0) [31]. It was similar to previously reported findings [32]. Forty-two and 78 patients had positive and negative CSF profiles of AD pathology, respectively.

### Biomarker quality control

Metabolites with >5% missingness or below limits of quantification were excluded, which left eight CSF and plasma metabolites (Table 1). Data were log_10_-transformed to tend toward a Gaussian distribution and standardized to null average and SD of 1 prior to statistical analyses. One low-quality CSF sample was not analyzed. CSF and plasma metabolite data were available for 119 and 120 subjects, respectively.

### Statistical analyses

Using least absolute shrinkage and selection operator (LASSO) logistic regression [33], we selected biomarkers predict both cognitive impairment and CSF profiles of AD pathology. A reference model was initially generated, testing variables that are likely to be available to the clinician and known risk factors for AD to provide a benchmark for comparison with the models that included one-carbon metabolites. These inputs included age, sex, years of education, presence of *APOE* ε4 allele, CSF Aβ_1–42_, CSF tau, and CSF p-tau181 concentrations for the prediction of cognitive impairment; and age, sex, years of education, and presence of *APOE* ε4 allele for the prediction of AD CSF profiles. In addition to all variables used to make the reference models, all metabolite measurements and CSF albumin index were then included in building so-called best models. The best models were built using LASSO regression by entering the CSF and plasma metabolites separately, thereby producing the two best models. A tenfold cross-validation process was performed for each LASSO analysis using the glmnet package in R [34], which allows estimating the confidence interval (CI) of the misclassification error for each value of the regularization parameter λ. The LASSO analyses were repeated 100 times (1000 times for the reference model). The models that minimized the upper limit of the cross-validated misclassification error CI across the 100 runs were selected. Their performance was assessed by ROC area under the curve (AUC) estimation using a bootstrap approach with 1000 iterations [35]. Results were compared visually and formally tested for significance against the reference model using ROC AUC [36] and accuracy using the McNemar test.

Two-sided correlation analyses between metabolites and CSF Aβ_1–42_, tau, and p-tau181 were performed with Pearson’s statistics and Bonferroni-corrected for multiple comparisons. Significant correlations between the metabolites and cofactors (*p* < 0.05) with CSF tau or p-tau181 were studied further in a linear regression model with tau or p-tau181 as the dependent variable and the following explanatory covariates: each metabolite, age, sex, *APOE* genotype (presence versus absence of an APOE ε4 allele), CDR (0 versus >0), and CSF albumin index (only for plasma metabolites). Values of the regression terms were reported, and their differences from 0 were assessed with *t* tests. Interaction terms between the metabolites and age, sex, CDR, or *APOE* genotype were identified in a type II analysis of variance (ANOVA) with an *F* test.

## Results

### Demographic and clinical characteristics of the study population

The cognitively impaired subjects were older and less educated and had a higher prevalence of the *APOE* ε4 genotype than the cognitively intact group (CDR 0) (Table 1). CSF Aβ_1–42_ was lower and CSF tau, CSF p-tau181, CSF p-tau181/Aβ_1–42_, and CSF albumin index were all higher in those with cognitive impairment. Concentrations of the one-carbon metabolites in CSF and plasma are provided in Table 1.

### Reference benchmark for the prediction of cognitive impairment

The reference model for classification of cognitive impairment included age, years of education, CSF Aβ_1–42_, CSF tau, and CSF p-tau181 and produced a diagnostic accuracy of 80.8% and an ROC AUC of 0.86 (95% CI 0.79–0.92). This model classified 59 of 72 cognitively impaired subjects correctly and 38 of 48 cognitively intact subjects correctly. By comparison, a majority class prediction [37] produced a diagnostic accuracy of about 60% to distinguish subjects with CDR >0 from subjects with CDR 0.

### CSF one-carbon metabolism and prediction of cognitive impairment

**Fig. 1:**
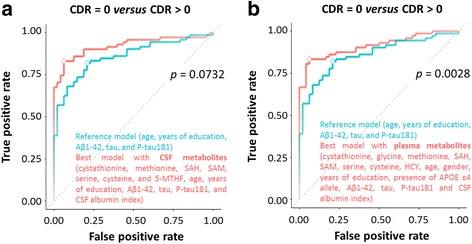
ROC curves of the (**a**) cerebrospinal fluid (CSF) and (**b**) plasma models predictive of cognitive impairment (Clinical Dementia Rating [CDR] 0 or >0). The CSF model includes cystathionine, methionine, *S*-adenosyl-L-homocysteine (SAH), *S*-adenosylmethionine (SAM), serine, cysteine, and 5-methyltetrahydrofolate (5-MTHF) in addition to age, years of education, CSF β-amyloid 1–42 peptide chain (Aβ_1–42_), CSF tau, CSF tau phosphorylated at threonine 181 (p-tau181), and CSF albumin index. The plasma model includes cystathionine, glycine, methionine, SAH, SAM, serine, cysteine, and homocysteine (Hcy) in addition to age, sex, years of education, presence of apolipoprotein E (*APOE*) ε4 allele, CSF Aβ_1–42_, CSF tau, CSF p-tau181, and CSF albumin index. The ROC curves of the least absolute shrinkage and selection operator (LASSO) models including metabolites (in *red*) are compared with the ROC curves of the reference LASSO models (in *blue*). The reference model is composed of age, years of education, CSF Aβ_1–42_, CSF tau, and CSF p-tau181. The opacity of the curves is proportional to the accuracy of the models. The *diamonds* indicate the selected most accurate models. The *p* values on the graphs indicate the significance of the differences of AUC. *APOE Apolipoprotein E, HCY Homocysteine, 5-MTHF 5-Methyltetrahydrofolate, SAH S-adenosyl-L-homocysteine, SAM S-adenosylmethionine*

After adding the CSF one-carbon metabolites, the diagnostic accuracy reached 87.4% (McNemar *p* value of 0.0704 compared with reference model), and the ROC AUC reached 0.92 (CI 0.87–0.96) (*p* = 0.0732 versus the reference model, as shown in Fig. 1a). The CSF metabolites included cystathionine, methionine, SAH, SAM, serine, cysteine, and 5-MTHF. Age, years of education, CSF Aβ_1–42_, CSF tau, CSF p-tau181, and CSF albumin index were also retained in the best model.

To evaluate the individual contribution of each metabolite selected in the best models, we conducted group comparisons for each metabolite. CSF methionine and serine were higher in cognitive impairment, with *p* values of 5.5 × 10^−6^ and 5.5 × 10^−3^, respectively (Table 1 and Additional file 1: Figure S1). We also explored other CSF metabolites not chosen in the best model and observed CSF SAH (*p* = 8.8 × 10^−3^) and choline (*p* = 0.01) elevation in the cognitively impaired patients.

### Plasma one-carbon metabolism and prediction of cognitive impairment

After adding the plasma one-carbon metabolites in the prediction of cognitive impairment, the diagnostic accuracy reached 87.5% (McNemar *p* value of 0.0614), and the ROC AUC was improved to 0.91 (CI 0.86–0.96) (*p* = 0.0028 versus the reference model, as shown in Fig. 1b). The best model included plasma cystathionine, glycine, methionine, SAH, SAM, serine, cysteine, and Hcy. Age, sex, years of education, presence of *APOE* ε4 allele, CSF Aβ_1–42_, CSF tau, CSF p-tau181, and CSF albumin index were in this best model. Plasma cysteine and SAH were elevated in cognitively impaired individuals, with *p* values of 5.5 × 10^−4^ and 4.6 × 10^−3^, respectively (Table 1 and Additional file 1: Figure S2).

### Reference benchmark for prediction of CSF profile of AD pathology

The reference model for classification of the CSF profile of AD pathology included age and presence of *APOE* ε4 allele. It had a diagnostic accuracy of 78.3% and an ROC AUC of 0.83 (CI 0.74–0.90). A majority class prediction produced a diagnostic accuracy of about 65% to distinguish subjects with p-tau181/Aβ_1–42_ ≤ 0.0779 from subjects with p-tau181/Aβ_1–42_ > 0.0779.

### CSF and plasma one-carbon metabolism and prediction of CSF profile of AD pathology

CSF and plasma one-carbon metabolites were both unable to improve the classification of CSF profile of AD pathology beyond the reference model (data not shown).

### One-carbon metabolites and AD pathology

**Fig. 2:**
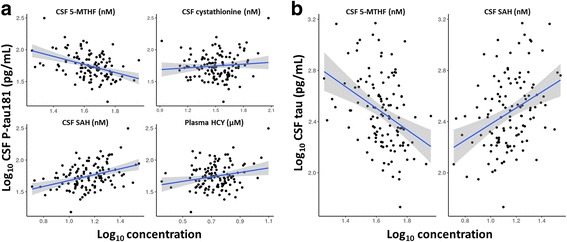
Cerebrospinal fluid (CSF) and plasma metabolites correlated with CSF tau phosphorylated at threonine 181 (p-tau181) (**a**) and CSF tau (**b**). Concentration values were log_10_-transformed. Each dot represents a subject. The *black line* represents the linear fit, and the *gray shading* represents its confidence interval. *HCY* Homocysteine, *5-MTHF* 5-Methyltetrahydrofolate, *SAH S*-adenosyl-L-homocysteine, *SAM S*-adenosylmethionine

CSF levels of Aβ_1–42_, tau, and p-tau181 were significantly different between CDR categories (Table 1). The correlations of individual metabolite levels in CSF and plasma with those markers of AD pathology were therefore assessed. None of the one-carbon metabolites correlated with CSF Aβ_1–42_. On the contrary, CSF cystathionine, SAH, and 5-MTHF as well as plasma Hcy were correlated with CSF p-tau181 (Fig. 2a). CSF SAH and 5-MTHF were significantly associated with CSF tau (Fig. 2b). CSF 5-MTHF was inversely correlated with both CSF p-tau181 and tau (Fig. 2).

**Fig. 3:**
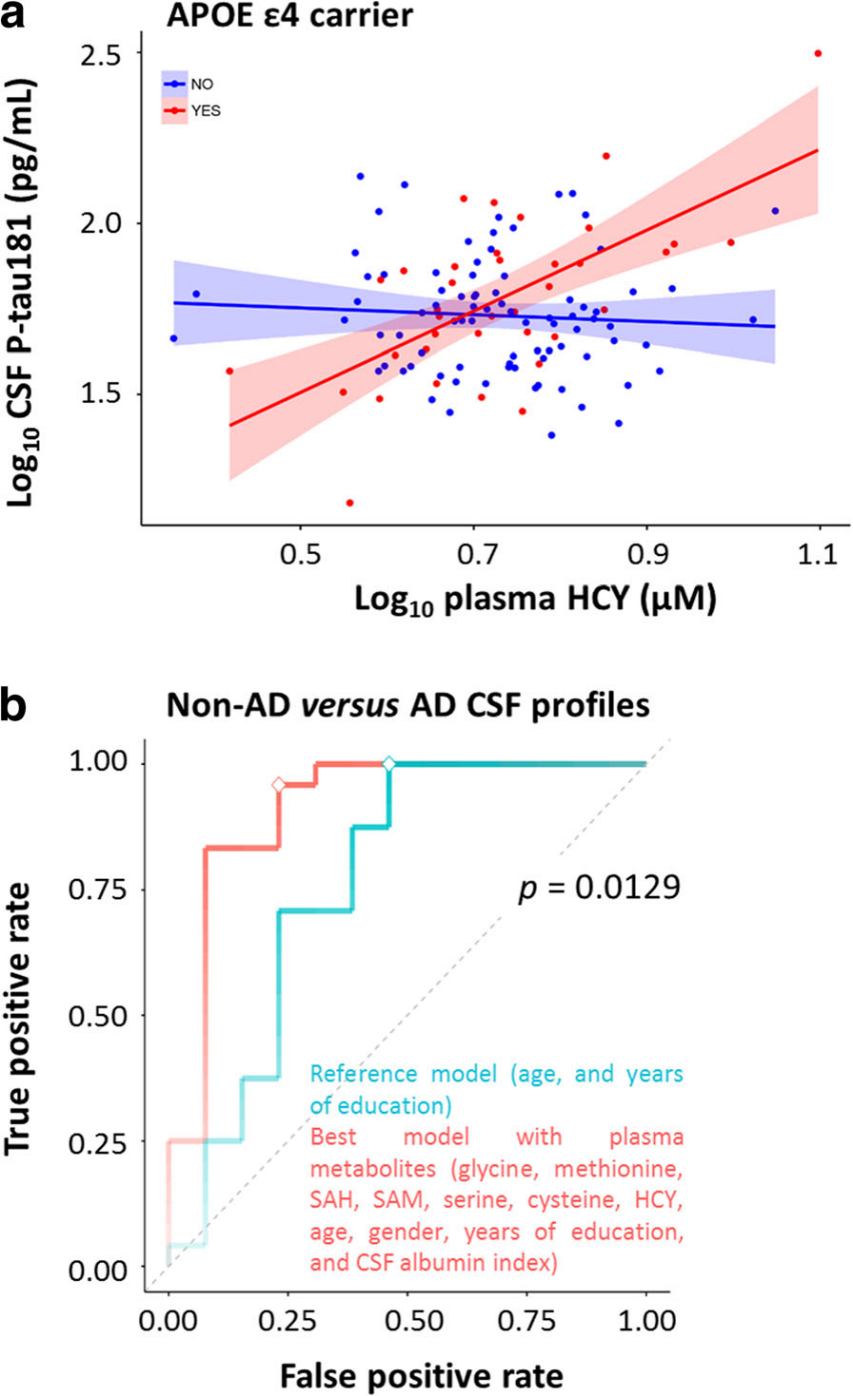
Association of homocysteine (Hcy) levels measured in plasma with cerebrospinal fluid (CSF) tau phosphorylated at threonine 181 (p-tau181), stratified by the presence of the apolipoprotein E (*APOE*) ε4 allele (**a**). The correlation between expression levels of plasma Hcy and CSF p-tau181 differs according to *APOE* genotype. ROC curves of the models predictive of non-Alzheimer disease (non-AD) versus AD CSF profiles (i.e., p-tau181/Aβ_1–42_ ≤ 0.0779 and p-tau181/Aβ_1–42_ > 0.0779, respectively) (**b**). The ROC curves of the least absolute shrinkage and selection operator (LASSO) models including metabolites in plasma (in *red*) are compared with the ROC curves of the reference LASSO models (in *blue*). The plasma model (in *red*) includes glycine, methionine, *S*-adenosyl-L-homocysteine (SAH), *S*-adenosylmethionine (SAM), serine, cysteine, and Hcy in addition to age, sex, years of education, and CSF albumin index. The reference model (in *blue*) includes age and years of education. The opacity of the curves is proportional to the accuracy of the models. The *diamonds* indicate the selected most accurate models. The *p* value on the graph indicates the significance of the differences of AUC. *APOE Apolipoprotein E, HCY Homocysteine, SAH S-adenosyl-L-homocysteine, SAM S-adenosylmethionine*

Metabolites significantly correlated with CSF p-tau181 or CSF tau were studied further in a linear regression model. The coefficients of each regression term and their significance are summarized in Table 2 for p-tau181 and tau. The cognitive impairment status term (CDR 0 versus CDR >0) was found to be associated in all models, confirming the association of high levels of CSF tau and CSF p-tau181 with cognitive impairment. Interaction terms between metabolite biomarkers and age, sex, CDR, and *APOE* ε4 genotype were then added to the regression models and tested by ANOVA (Table 3). An interaction was observed for CSF p-tau181 between plasma Hcy and *APOE* ε4 genotype (Fig. 3a). For CSF p-tau181, significant interactions were also obtained between SAH and 5-MTHF in CSF and *APOE* ε4 genotype. Sex was found to interact with CSF SAH and plasma Hcy for CSF p-tau181 and with CSF SAH for CSF tau.

**Table 2:** Regression coefficients in linear model with cerebrospinal fluid tau phosphorylated at threonine 181 and tau as dependent variables

Regression term	CSF cystathionine	CSF SAH	CSF 5-MTHF	Plasma Hcy
CSF p-tau181				
Intercept	1.0719^a^	1.0503^a^	2.1744^a^	1.1802^a^
Age	0.0063^b^	0.0038	0.0045^c^	0.0054^c^
Sex^d^	−0.0279	−0.0223	−0.0140	−0.0236
*APOE* ε4 genotype^e^	0.0328	0.0195	0.0362	0.0314
CDR category^f^	0.1173^b^	0.1182^b^	0.1110^b^	0.1186^b^
Biomarker	0.1120	0.3195^b^	−0.4965^a^	0.1670
CSF tau				
Intercept		1.4134^a^	2.8174^a^	
Age		0.0073^c^	0.0080^c^	
Sex^d^		−0.0215	−0.0108	
*APOE* ε4 genotype^e^		0.0692	0.0894	
CDR category^f^		0.1758^a^	0.1665^b^	
Biomarker		0.3803^b^	−0.6269^a^	

*Abbreviations: Aβ* β-Amyloid, *APOE* Apolipoprotein E, *CDR* Clinical Dementia Rating, *CSF* Cerebrospinal fluid, *Hcy* Homocysteine, *5-MTHF* 5-Methyltetrahydrofolate, *p-tau181* Tau phosphorylated at threonine 181, *SAH S*-adenosyl-L-homocysteine

Values of the regression terms are reported, and their differences from 0 were assessed with a *t* test

^a^ *p* ≤ 0.001

^b^ *p* ≤ 0.01

^c^ *p* ≤ 0.05

^d^Females (versus reference males)

^e^ *APOE* ε4 carriers (versus reference noncarriers)

^f^ CDR >0 (versus reference CDR 0)

**Table 3:** Regression coefficients in linear model with cerebrospinal fluid tau phosphorylated at threonine 181 and tau as dependent variables of interaction terms between biomarkers and different variables

Interaction term	CSF cystathionine	CSF SAH	CSF 5-MTHF	Plasma Hcy
CSF p-tau181				
Age	−0.0011	−0.0098	0.0036	0.0113
Sex^a^	−0.0652	−0.4815^b^	0.3443	−0.6850^b^
*APOE* ε4 genotype^c^	0.2169	0.5126^b^	−0.5687^b^	1.1427^d^
CDR category^e^	0.1877	0.2122	−0.2434	0.2833
CSF tau				
Age		−0.0076	0.0099	
Sex^a^		−0.7407^f^	0.4541	
*APOE* ε4 genotype^c^		0.4953	−0.6965	
CDR category^e^		0.1352	−0.2681	

*Abbreviations: APOE* Apolipoprotein E, *CDR* Clinical Dementia Rating, *CSF* Cerebrospinal fluid, *Hcy* Homocysteine, *5-MTHF* 5-Methyltetrahydrofolate, *p-tau181* Tau phosphorylated at threonine 181, *SAH S*-adenosyl-L-homocysteine

Every interaction was tested in separate models, with only one interaction per model. Values of the regression interaction terms are reported, and their difference from 0 was assessed with an *F* test in analysis of variance

^a^Females (versus reference males)

^b^ *p* ≤ 0.05

^c^ *APOE* ε4 carriers (versus reference noncarriers)

^d^ *p* ≤ 0.001

^e^CDR >0 (versus reference CDR 0)

^f^ *p* ≤ 0.01

### Interaction of *APOE* ε4 genotype in one-carbon metabolite-based prediction of AD pathology

Because we observed some metabolite interactions with *APOE* genotype, we stratified the analysis on the basis of individuals carrying the *APOE* ε4 allele (*n* = 37). In this subgroup, no correlation was found with CSF levels of Aβ_1–42_ and individual metabolite levels in both CSF and plasma. Cystathionine, SAH, and 5-MTHF in CSF, as well as Hcy in plasma, showed significant correlations with CSF p-tau181 (Additional file 1: Figure S3a). Concentrations of SAH and 5-MTHF in CSF and Hcy in plasma were significantly associated with CSF tau (Additional file 1: Figure S3b). Again, however, except for cognitive impairment status (CDR 0 versus CDR >0), these observed correlations were not impacted by other clinical covariates (data not shown).

The metabolite measurements were considered in a LASSO logistic regression to classify patients with non-AD versus AD CSF profiles in this subgroup of individuals. A reference LASSO logistic regression on known or probable contributing variables (i.e., age and years of education) provided a reference model with an accuracy of 83.8% and an ROC AUC of 0.76 (CI 0.55–0.92). Here, a majority class classifier provided 64.9% diagnostic accuracy. Including metabolite markers measured in CSF did not improve the ability to predict an AD biomarker profile compared with the reference model. In contrast, plasma metabolites provided nonsignificant improvement in terms of accuracy but significantly increased performance for AUC (Fig. 3b). The model with plasma metabolites presented an accuracy of 89.2% (McNemar *p* value of 0.6831) and an ROC AUC of 0.92 (CI 0.7–1.00) (*p* = 0.0129 versus the reference model). Seven plasma metabolites were included in this mathematical model (i.e., glycine, methionine, SAH, SAM, serine, cysteine, and Hcy) in addition to age, sex, years of education, and CSF albumin index. Box plots of plasma metabolite measurements for non-AD versus AD CSF profiles in *APOE* ε4 carriers are provided in Additional file 1: Figure S4.

## Discussion

In the present study, we deployed a LC-MS/MS-based method to expand previous work [38], targeting one-carbon metabolism in both CSF and plasma, and to better understand its role in cognitive impairment and AD pathology. We identified CSF and plasma profiles including several one-carbon metabolites beyond Hcy associated with cognitive impairment in older adults. The association between several CSF and plasma one-carbon metabolites with CSF tau and p-tau181 warrants further research. Although we were unable to predict an a priori-stated CSF profile of AD pathology in the total cohort, the plasma one-carbon metabolites markedly improved the classification of CSF profiles of AD pathology in APOE ε4 carriers.

Our results highlight the contribution of methionine, serine, choline, and cysteine in addition to the more commonly assessed and reported one-carbon metabolites (i.e., SAM, SAH, and Hcy). Methionine supplementation in wild-type mice can induce neurotoxicity, higher levels of tau phosphorylation and Aβ peptides in the brain, and memory loss [39]. These mechanisms may explain our observation of higher CSF methionine in cognitive impairment. Protective effects of choline against age-related cognitive deficits were previously evidenced in animal models [40], and better memory performance was related to a higher concurrent choline intake in a large, nondemented, community-based human population [41]. However, we observed higher CSF choline in cognitive impairment, an inconsistency that may reflect neurodegeneration and the breakdown of synaptic membranes enriched with choline [42]. We showed significantly increased CSF and plasma SAH in cognitive impairment, which is consistent with previous findings [14, 43].

Several metabolites of the one-carbon cycle measured in CSF and plasma were associated with tau metabolism, reflected by total tau and p-tau181 in the CSF, after controlling for potential confounders. Higher CSF 5-MTHF appeared protective, whereas higher CSF SAH appeared to promote tau aggregation and neuronal injury. These results are consistent with reports demonstrating higher CSF SAH and lower CSF 5-MTHF associated with higher CSF p-tau181 [14]. Accordingly, impairment of the one-carbon metabolism, induced by feeding with a high-methionine/low-folate diet, was shown to increase SAH levels, downregulate methyltransferase, reduce protein phosphatase 2A methylation, and induce accumulation of neurofibrillary tangle pathology in mice [44]. Higher levels of tau phosphorylation were also observed in the brains of wild-type mice fed a methionine-enriched diet, putatively inducing the fragmentation of tau from microtubules and leading to fibrillization [39]. Together, these results suggested that altered one-carbon metabolism may contribute to microtubule-associated tau protein hyperphosphorylation and neurodegeneration in general. Our inability to observe a relationship between any one-carbon metabolites with CSF Aβ_1–42_ was remarkable and consistent with some studies [14, 45], but in contradiction with other in vitro and in vivo preclinical investigations [46, 47]. A recent study of human adults with normal cognition found that disturbed one-carbon metabolism may be related to increased CSF levels of Aβ_1–42_ and soluble amyloid precursor protein forms, suggesting a contribution to the accumulation of cerebral amyloid pathology [48], making it difficult to draw conclusions at this time.

We were unable to predict CSF profiles of AD pathology until an interaction with *APOE* genotype was identified. For example, whereas plasma Hcy appeared insubstantial to CSF tau in the total sample, hyperhomocysteinemia was relevant to CSF tau in the *APOE* ε4 carriers. This was further corroborated by our prediction of positive AD pathology using the p-tau181/Aβ_1–42_ ratio after restricting the analysis to *APOE* ε4 carriers only. Although this interaction between Hcy, *APOE*, and tau pathology was clear in our analysis, the biological rationale behind this interaction is less clear. A recent report demonstrated that plasma Hcy predicts the conversion from MCI to AD only in *APOE* ε4 carriers [49], adding a further level of consistency to our findings. Other metabolites of the one-carbon cycle, such as CSF SAM [50], may also be more relevant in *APOE* ε4 carriers and should be further explored.

Taken together, our results show that one-carbon metabolism is relevant to cognition independent of and beyond AD core pathology. One-carbon metabolism disturbances may cripple DNA repair, methylation, and/or synthesis, as well as reduce the availability of neurotransmitters, phospholipids, and myelin [51]. Several of these mechanisms may be involved in cognitive dysfunction and underlying neurobiology.

The present study has limitations. We used measures of cognition and CSF measures of AD in a cross-sectional analysis, and the temporality of the associations cannot be extrapolated. Nevertheless, we present the most comprehensive mapping of the one-carbon pathway in both CSF and plasma in older adults. The results are encouraging and in the future should be replicated in an independent cohort as well as confirmed in relation to longitudinal change in cognition and the incidence of AD.

## Conclusions

Our results suggest that several one-carbon metabolites beyond Hcy are relevant to AD pathology and cognitive function in older adults. This may partially explain why Hcy-lowering trials have been mostly disappointing and opens the possibility that other metabolites in the pathway may also be relevant targets for the prevention of cognitive decline and the underlying neurobiology of AD.

## Additional file

Additional file 1: Figure S1. Box plots of CSF metabolite measurements for CDR of 0 and CDR >0 (*i.e.*, CDR 0.5 or 1). Level of significance is indicated as *p* value after Bonferroni correction. Values were log-transformed. **Figure S2.** Box plots of plasma metabolite measurements for CDR 0 and CDR >0 (*i.e.*, CDR 0.5 or 1). Level of significance is indicated as *p* value after Bonferroni correction. Concentration values were log-transformed. **Figure S3.** CSF and plasma metabolites correlated with CSF p-tau181 (**a**) and CSF tau (**b**) in *APOE* ε4 carriers (*n* = 37). Concentration values were log-transformed. Each dot represents a subject. The black line represents the linear fit, and the gray shading represents its confidence interval. **Figure S4.** Box plots of plasma metabolite measurements in *APOE* ε4 carriers (*n* = 37) according to CSF p-tau181/Aβ_1–42_ ratio (*i.e.*, “low” when p-tau181/Aβ_1–42_ is ≤0.0779 and “high” when p-tau181/Aβ_1–42_ is > 0.0779) for negative and positive CSF profiles of AD pathology, respectively. Concentration values were log_10_-transformed. Level of significance is indicated as *p* value after Bonferroni correction. **Supplementary Methods**. (PDF 831 kb)

## Abbreviations

Aβ: β-Amyloid
AD: Alzheimer disease
ANOVA: Analysis of variance
*APOE*: Apolipoprotein E
CDR: Clinical Dementia Rating
CSF: Cerebrospinal fluid
Hcy: Homocysteine
LASSO: Least absolute shrinkage and selection operator
LC-MS/MS: Liquid chromatography-tandem mass spectrometry
MCI: Mild cognitive impairment
MMSE: Mini Mental State Examination
5-MTHF: 5-Methyltetrahydrofolate
p-tau181: Tau phosphorylated at threonine 181
SAH: *S*-adenosyl-L-homocysteine
SAM: *S*-adenosylmethionine
