# Supplementary material for: One-carbon metabolism, cognitive impairment and CSF measures of Alzheimer pathology: homocysteine and beyond
Source: Alzheimers Res Ther. 2017 Jun 17;9:43. doi: 10.1186/s13195-017-0270-x (PMC5473969; doi:10.1186/s13195-017-0270-x)
Supplement: Additional file 1: Figure S1. — Box plots of CSF metabolite measurements for CDR of 0 and CDR >0 (i.e., CDR 0.5 or 1). Level of significance is indicated as p value after Bonferroni correction. Values were log-transformed. Figure S2. Box plots of plasma metabolite measurements for CDR 0 and CDR >0 (i.e., CDR 0.5 or 1). Level of significance is indicated as p value after Bonferroni correction. Concentration values were log-transformed. Figure S3. CSF and plasma metabolites correlated with CSF p-tau181 (a) and CSF tau (b) in APOE ε4 carriers (n = 37). Concentration values were log-transformed. Each dot represents a subject. The black line represents the linear fit, and the gray shading represents its confidence interval. Figure S4. Box plots of plasma metabolite measurements in APOE ε4 carriers (n = 37) according to CSF p-tau181/Aβ1–42 ratio (i.e., “low” when p-tau181/Aβ1–42 is ≤0.0779 and “high” when p-tau181/Aβ1–42 is > 0.0779) for negative and positive CSF profiles of AD pathology, respectively. Concentration values were log10-transformed. Level of significance is indicated as p value after Bonferroni correction. Supplementary Methods. (PDF 831 kb) [file 13195_2017_270_MOESM1_ESM.pdf]

ADDITIONAL FILE

One-carbon metabolism, cognitive impairment and CSF measures of Alzheimer pathology: Homocysteine and beyond

Loïc Dayon[1, *], Seu Ping Guiraud[1], John Corthésy[1], Laeticia Da Silva[1], Eugenia Migliavacca[1], Domilė Tautvydaitė[2], Aikaterini Oikonomidi[2], Barbara Moullet[2], Hugues Henry[3], Sylviane Métairon[1], Julien Marquis[1], Patrick Descombes[1], Sebastiano Collino[1], François-Pierre J. Martin[1], Ivan Montoliu[1], Martin Kussmann[1, $], Jérôme Wojcik[4], Gene L. Bowman[1], and Julius Popp[2]

[1]Nestlé Institute of Health Sciences, Lausanne, Switzerland

[2]CHUV, Old Age Psychiatry, Department of Psychiatry, Lausanne, Switzerland

[3]CHUV, Department of Laboratories, Lausanne, Switzerland

[4]Quartz Bio, Geneva, Switzerland

[$]Current affiliation: Liggins Institute, University of Auckland, New Zealand

Correspondence to: *Nestlé Institute of Health Sciences, EPFL Innovation Park, Bâtiment H, 1015 Lausanne, Switzerland; Email: loic.dayon@rd.nestle.com, Phone: +41 21 632 6114, Fax: +41 21 632 6499

Figure S1. Box-plots of CSF metabolite measurements for CDR = 0 and CDR > 0 (*i.e.*, CDR = 0.5 or 1). Level of significant is indicated as *p*-value after Bonferroni correction. Values were log-transformed.

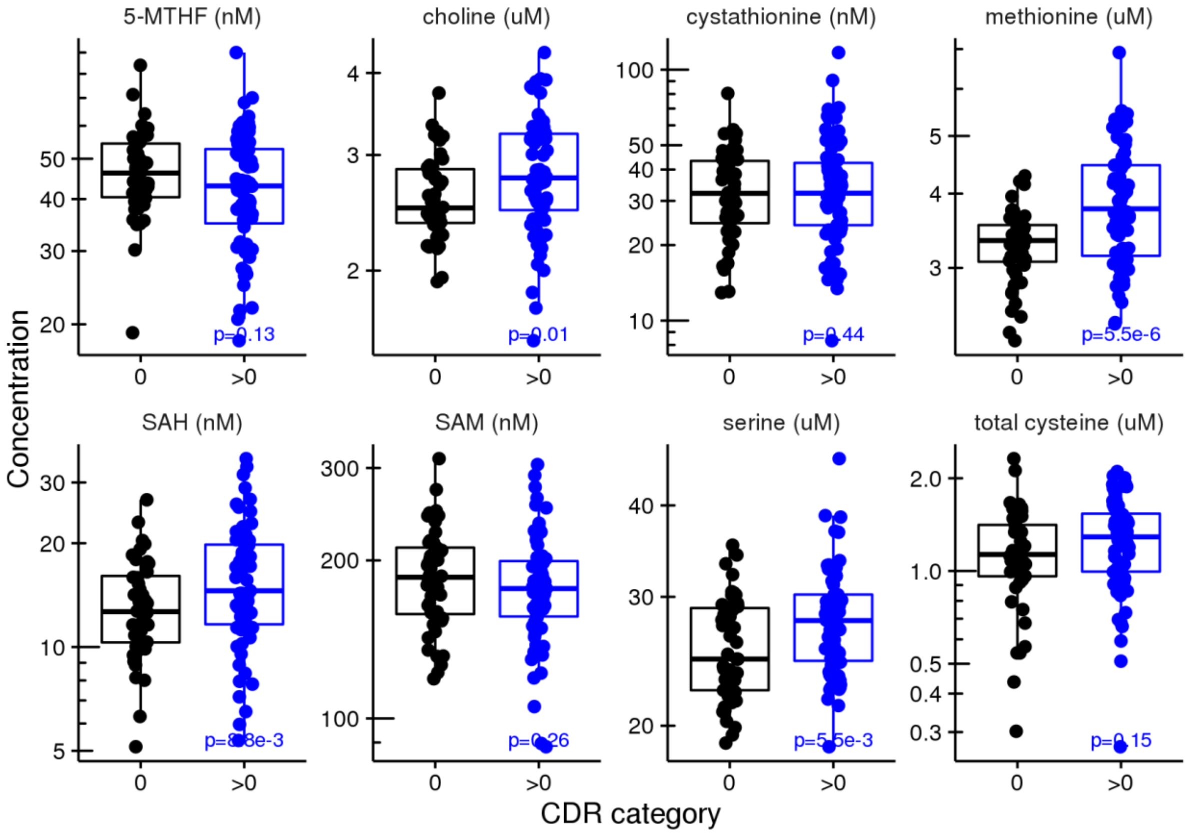

Figure S2. Box-plots of plasma metabolite measurements for CDR = 0 and CDR > 0 (*i.e.*, CDR = 0.5 or 1). Level of significant is indicated as *p*-value after Bonferroni correction. Concentration values were log-transformed.

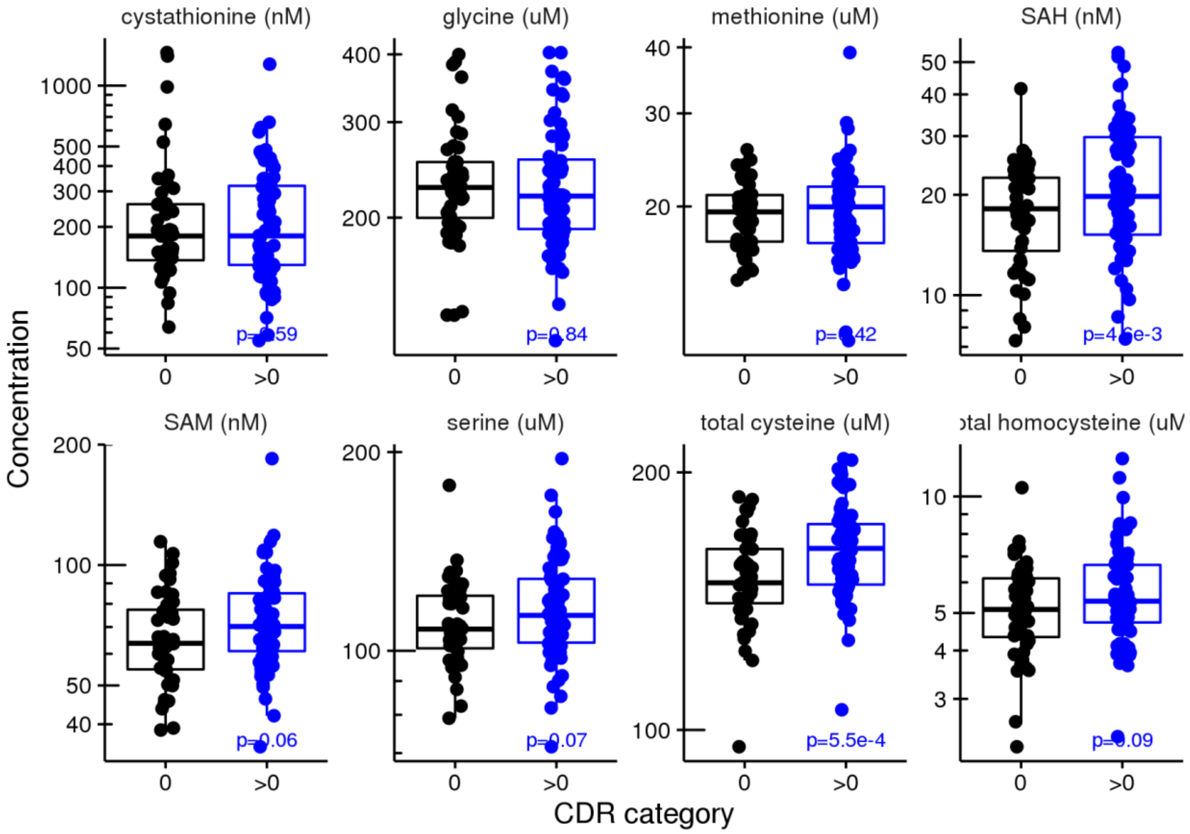

Figure S3. CSF and plasma metabolites correlated with CSF P-tau181 (a) and CSF tau (b) in APOE ε4 carriers (n = 37). Concentration values were log-transformed. Each dot represents a subject. The black line represents the linear fit and the gray shading its confidence interval.

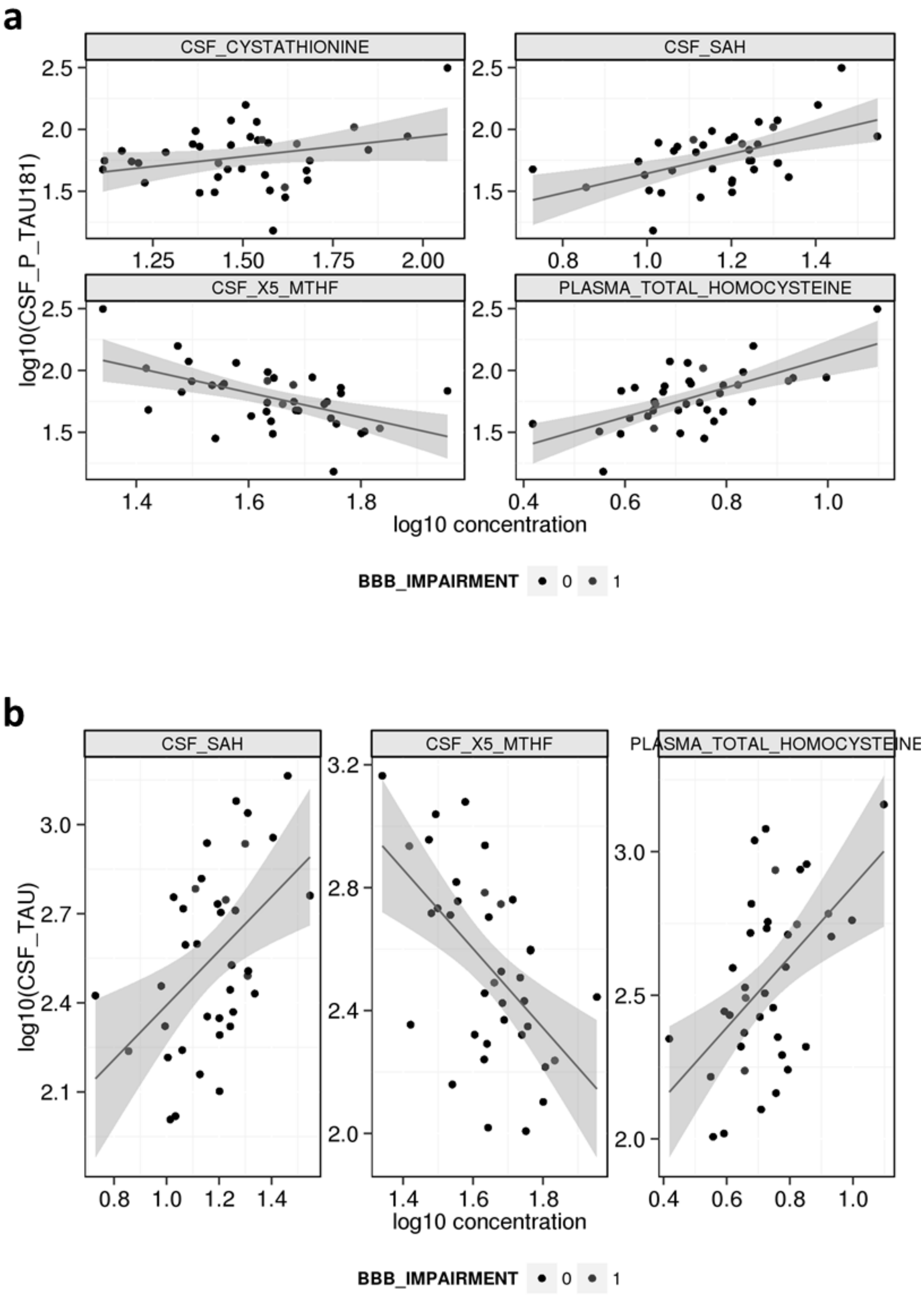

Figure S4. Box-plots of plasma metabolite measurements in APOE ε4 carriers (*n* = 37) according to CSF P-tau181/Aβ1-42 ratio, *i.e.*, "low" when P-tau181/Aβ1-42 ≤ 0.0779 and "high" when P-tau181/Aβ1-42 > 0.0779 for negative and positive CSF profiles of AD pathology respectively. Concentration values were $\log_{10}$-transformed. Level of significant is indicated as *p*-value after Bonferroni correction.

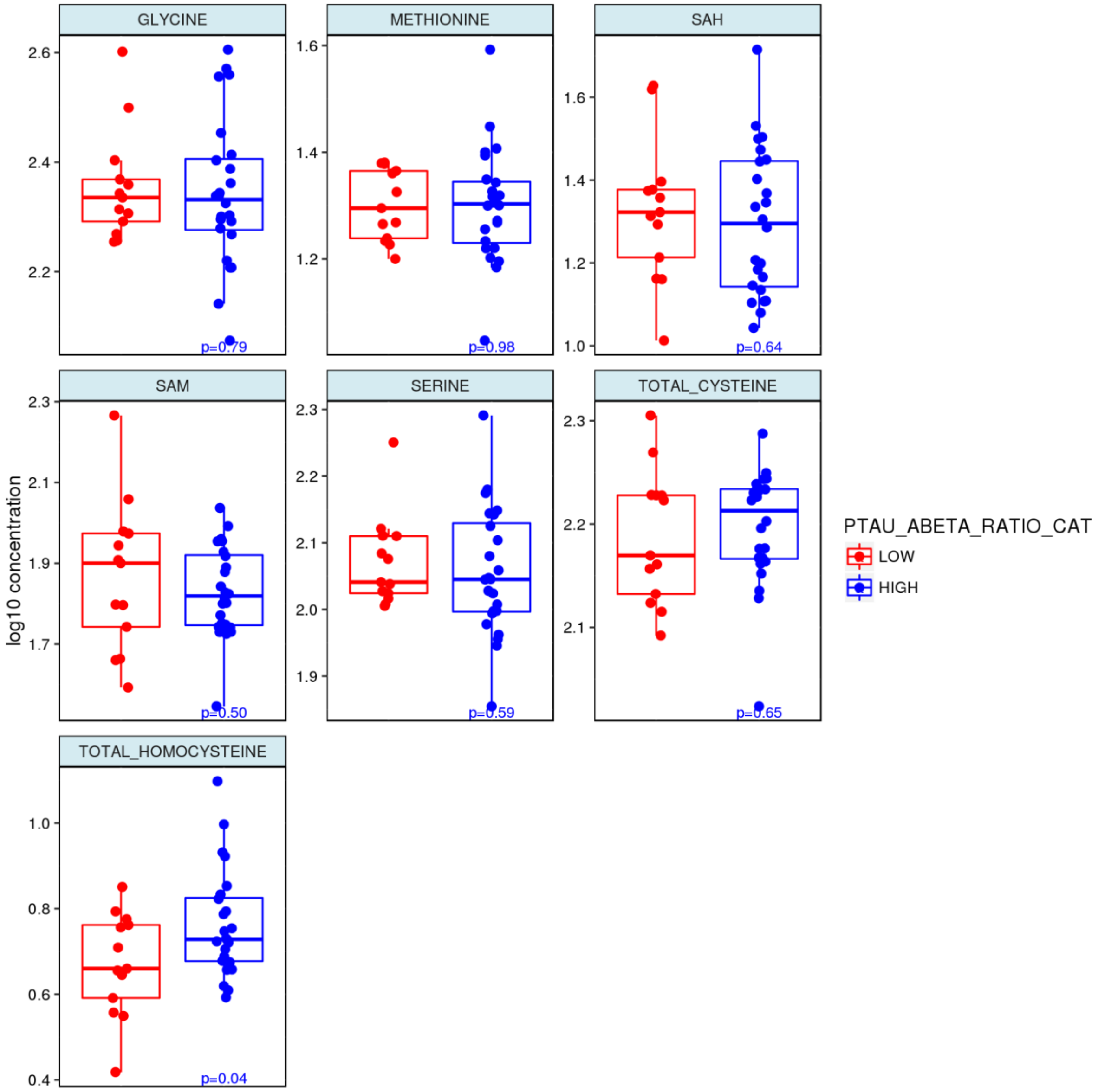

SUPPLEMENTARY METHODS

Chemical reagents. LC-MS grade acetonitrile (CH_3CN), LC grade methanol (MeOH), formic acid (FA), trichloroacetic acid (TCA), perfluorohepatanoic acid (PFHA), ascorbic acid (AA), sodium hydroxide (NaOH) 0.1 M, hydrochloric acid (HCl), tris(2-carboxyethyl)phosphine (TCEP), dithiothreitol (DTT) and ammonium acetate (NH_4OAc) were purchased from Sigma-Aldrich (Buchs, Switzerland). Deionized water (H_2O) (18.2 MΩ·cm at 25 °C) was obtained from a Milli-Q apparatus (Millipore, Bedford, MA, USA). Homocysteic acid (HA), taurine, serine, cystine, glycine, homocystine (HCY2), riboflavin, methionine, pyridoxine, cystathionine, S-adenosylhomocysteine (SAH), pyridoxamine, S-adenosylmethionine (SAM), dimethylglycine (DMG), choline, betaine, 5-methyltetrahydrofolic acid (5-MTHF), taurine-$^{13}C_2$, glycine-D_2, riboflavin-dioxopyrimidine-$^{13}C_4{}^{15}N_2$ and methionine-D_3 standards were purchased from Sigma-Aldrich. HA-D_4, serine-D_3, pyridoxine-D_2, cystathionine-D_4, SAM-D_4, DMG-D_6, choline-D_9, betaine-D_{11} were purchased from CDN Isotopes (Pointe-Claire, Quebec, Canada). Cystine-D_4, HCY2-D_8 and pyridoxamine-D_3 were from Cambridge Isotopes Laboratories (Andover, M.A., USA) and SAH-D_4 was from Cayman Chemical (Ann Arbor, MI, USA). 5-MTHF-$^{13}C_5$ was purchased from Merck (Schaffhausen, Switzerland).

Targeted metabolomics of the one-carbon cycle. Light and heavy standards were first dissolved individually in 0.1 M HCl (or in 0. 1 M NaOH, 10 mmol·L^{-1} NH_4OAc or in a mixture of MeOH/H_2O depending on their solubility). Calibration and internal standard (IS) solutions were prepared by dilution of the standard stock solutions in CH_3CN/H_2O (95%/5%). Calibration solutions were made according to the concentration ranges indicated in **Table 1** for each body fluid; they were freshly prepared with each batch of samples and injected at the beginning and the end of each batch. Quality controls (QCs) at two different concentrations were added to each sample

batch. Frozen samples were thawed out at room temperature and vortexed for 10 s. Volumes of 50 µL of CSF or plasma samples were transferred into 1.5 mL microcentrifuge tubes. A volume of 10 µL of IS solution was added to CSF or plasma, then 50 µL of a TCEP solution followed by 140 µL of MeOH with 1% FA. The tubes were vortexed for 15 min at 1400 rpm (4 °C) and centrifuged at 14500 rpm for 5 min. The supernatants were pipetted and filtered through a 0.22 µm filter before LC-MS/MS analysis. LC-MS/MS was performed with an Accela UHPLC 1250 Pump coupled to a TSQ Quantum Vantage triple quadrupole mass spectrometer equipped with a heated electrospray ionization (H-ESI) source (Thermo Scientific, San Jose, CA, USA). LC separation was performed using a gradient elution on a reversed-phase (RP) Acquity UPLC HSST3 1.8 µm, 100 × 2.1 mm column (Waters, Milford, MA, USA) at a flow rate of 400 $\mu L \cdot min^{-1}$. Mobile phase A was H_2O containing 5 mM PFHA and mobile phase B was CH_3CN. The gradient profile was: (a) 0 min 95% A; (b) 0.5 min 95% A; (c) 8 min 5% A; (d) 10 min 5% A; (e) 11 min 95% A; (f) 13 min 95% A. The injection volume was 10 µL and the total run time was 13 min. MS detection was performed in positive electrospray ionization at unit resolution. Electrospray voltage, sheath gas, auxiliary gas, vaporizer temperature, and capillary temperature were respectively 4 kV, 10 arbitrary units, 5 arbitrary units, 200 °C and 350 °C. The collision gas (*i.e.*, argon) was set at 1.5 mTorr. Selected reaction monitoring (SRM) transitions have been described previously [23]. Automated tuned s-lens parameters were used and the scan time was set to 0.02 s. Xcalibur 2.1 software (Thermo Fisher Scientific) was used for instrument control, data acquisition and processing.

CSF Aβ1-42, tau, and P-tau181. CSF Aβ1-42, total-tau (tau), and tau phosphorylated at threonine 181 (P-tau181) concentrations were measured using commercially available ELISA kits (Fujirebio, Ghent, Belgium).

APOE genotyping. DNA was extracted from whole blood using the QIAsymphony DSP DNA Kit (Qiagen, Hombrechtikon, Switzerland). The SNV rs429358 and rs7412 were genotyped using the Taqman assays C___3084793_20 and C____904973_10 respectively (Thermo Fischer Scientific, Waltham, MA USA).

REFERENCES

23. Guiraud SP, Montoliu I, Da Silva L, Dayon L, Núñez Galindo A, Corthésy J, et al. High-throughput and simultaneous quantitative analysis of homocysteine–methionine cycle metabolites and co-factors in blood plasma and cerebrospinal fluid by isotope dilution LC–MS/MS. Anal Bioanal Chem. 2017;409:295-305.
